# Comprehensive Flavor Profiling of Dairy Products Using Electronic Tongue: Discrimination Based on Processing Parameters and Formulations

**DOI:** 10.1002/fsn3.71781

**Published:** 2026-05-13

**Authors:** Juan Huang, Jing‐wei Zhong, Li‐guang Jiang, Yan‐yan Huang, Dong‐mei Liu

**Affiliations:** ^1^ School of Food and Pharmaceutical Science and Technology Guangzhou College of Technology and Business Guangzhou Guangdong China; ^2^ School of Food Science and Engineering South China University of Technology Guangzhou Guangdong China; ^3^ College of Food Science and Engineering Foshan University Foshan China; ^4^ Guangdong Provincial Key Laboratory of Intelligent Food Manufacturing Foshan University Foshan China

**Keywords:** dairy products, electronic tongue, flavor profiling, multivariable interaction, partial least squares regression, principal component analysis (PCA)

## Abstract

Flavor is a critical determinant of consumer acceptance for dairy products. However, objectively deconvoluting the contributions of multifactor interactions—such as processing conditions, formulations, and raw material properties—remains challenging. This study employed the INSENT TS‐5000Z electronic tongue (E‐tongue), supplemented by sensory evaluation and chemical analysis, to profile eight fresh milk and six plain yoghurt samples from five leading brands. Key variables included sterilization methods, milk sources, and sugar content. Nine taste indices were quantified. Principal component analysis (PCA) effectively discriminated samples based on these parameters, with cumulative variances of 81.2% for milk and 92.5% for yoghurt. Organic milk (SY‐JZ‐ORG) exhibited significantly higher bitterness (10.77 ± 0.04 TU) and astringency (4.60 ± 0.02 TU), which correlated with elevated levels of α‐linolenic acid, hydrophobic peptides, and the creamy lactone δ‐decalactone. Full‐sugar yoghurt (MN‐Y) showed pronounced astringency (9.38 ± 0.20 TU), consistent with its highest sensory scores for “thickness” and “astringency.” Partial least squares regression (PLSR) models reliably predicted sensory attributes from E‐tongue data (R^2^Y = 0.971–0.972, Q^2^ = 0.856–0.902), identifying astringency as the primary predictor. This study demonstrates that the E‐tongue can objectively decode complex flavor differences arising from multifactor interactions, offering a robust framework for dairy quality control and product development.

## Graphical abstract

The E‐tongue combined with sensory and chemical analysis effectively discriminated fresh milk and yoghurt based on processing parameters and formulations. Astringency emerged as the key predictor for quality control.

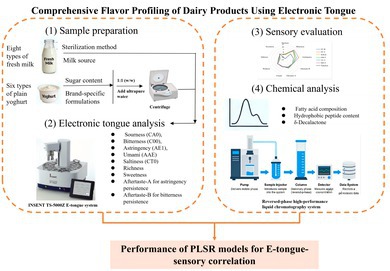

## 1 Introduction

Dairy products, particularly fresh milk and yoghurt, are globally consumed staple foods valued for their high nutritional density (e.g., complete proteins, calcium) and diverse sensory properties (Fox et al. 2015). Consumer preference and market competitiveness of dairy products are largely driven by flavor, which is shaped by interactive multifactors: raw material quality (e.g., organic vs. conventional milk, A2β‐casein vs. regular milk) (Nascimento et al. 2023; Choi et al. 2024; Ilić et al. 2024), processing techniques (e.g., pasteurization vs. ultra‐instant sterilization) (Zhang et al. 2025; Jiang et al. 2024), and formulation (e.g., sugar content, probiotic addition) (Kamali et al. 2023; Cutrim et al. 2023; Xi et al. 2023).

Traditional sensory evaluation, while intuitive, is inherently subjective—susceptible to panelist fatigue, individual taste variability, and environmental interference (Sipos et al. 2021; Stone and Sidel 2004)—limiting its application in high‐throughput quality control or precise flavor differentiation. Conventional chemical analysis (e.g., GC–MS) can quantify individual compounds but fails to capture holistic taste perception, as it does not account for synergistic or antagonistic interactions between taste‐active components.

Electronic tongue (E‐tongue) technology has emerged as a powerful alternative to overcome these limitations. By using cross‐sensitive sensor arrays, E‐tongues simulate human taste buds to capture comprehensive “taste fingerprints” (Calvini and Pigani 2022; Gharibzahedi et al. 2022; Kossakowska et al. 2022).

The INSENT TS‐5000Z system, a potentiometric electronic tongue employing artificial lipid membrane sensors, detects taste substances via electrostatic and hydrophobic interactions with the membrane, causing a change in membrane potential that is quantitatively measured. This enables the assessment of five basic tastes (sourness, sweetness, bitterness, saltiness, umami) and astringency—without requiring complex premodeling (Zhang et al. 2015). Its advantages include high correlation with human taste perception and straightforward operation; however, limitations include sensitivity to matrix effects from fats and proteins and an inability to detect volatile aroma compounds (Wu et al. 2020; Kobayashi et al. 2010).

The applicability of E‐tongue technology in dairy research has been widely demonstrated. Pan et al. (2019) showed that an E‐tongue with a CHEMFET sensor array could effectively differentiate skim milk subjected to various preheating conditions, with outputs highly correlated with human sensory scores. Schlossareck and Ross (2019) confirmed its capability to quantify “spiciness” in cheese, revealing a significant positive correlation with human panel assessments. Heema and Gnanalakshmi (2022) reviewed its utility for rapid evaluation of fundamental tastes in milk, yoghurt, and cheese. In more complex matrices, Cho and Moazzem (2022) demonstrated that the E‐tongue can sensitively discriminate between “umami” and “bitterness” in peptide‐rich dairy products, suggesting its potential to partially replace human panels for specific attributes.

Despite these advances, most previous studies have focused on single‐variable investigations—such as distinguishing UHT milk brands (Ciosek 2016) or monitoring flavor changes in yoghurt during storage (Hoxha et al. 2023). Systematic investigations of multifactor interactions (e.g., the combined effects of sterilization technique and milk source in fresh milk, or sugar content and brand in yoghurt) on flavor profiles remain scarce. Furthermore, a significant limitation of prior research is the frequent lack of direct chemical evidence (e.g., fatty acid profiles, peptide content) to mechanistically explain E‐tongue sensor responses, leaving the interpretation of flavor differences largely empirical.

In contrast to prior studies that examined single variables, this work systematically investigates multi‐factor interactions—such as sterilization method combined with milk source—and validates E‐tongue findings through chemical and sensory analyses, specifically aiming to: (1) Quantify complete taste profiles of fresh milk and plain yoghurt using the INSENT TS‐5000Z E‐tongue; (2) Identify key taste indices discriminating samples based on processing parameters and formulations, supported by chemical and sensory data; (3) Validate E‐tongue results with multivariate statistical models (PCA, PLSR) and cross‐validation.

## 2 Materials and Methods

### 2.1 Sample Preparation

#### 2.1.1 Sample Selection and Sampling Method

Eight fresh milk and six plain yoghurt samples from five major brands (Sanyuan, Junlebao, Yili, Mengniu, Guangming) were procured from Beijing supermarkets in June 2024. Samples were selected to represent key variables: for milk—sterilization method (pasteurization vs. UHTST) and milk source (organic, A2β‐casein, conventional); for yoghurt—sugar content (full‐sugar, reduced, sugar‐free) and brand‐specific formulations (probiotics, protein content). All samples were stored at 4°C and analyzed within 3 days. Detailed sample information is summarized in Table 1.

**TABLE 1:** Key characteristics of the dairy samples analyzed (*N =* 4 batches per sample).

Sample code	Brand	Details	Packaging	Batch no.	Key characteristics
Product: Fresh milk					
SY‐milk	Sanyuan	Full‐fat pasteurized (72°C, 15 s), 7‐day shelf life, 3.8% protein	Bottle	20240601	Conventional pasteurization, standard milk source
SY‐JZ‐ORG	Sanyuan Jizhi	Premium full‐fat pasteurized (72°C, 15 s), 7‐day shelf life, 3.5% protein	Bottle	20240602	Organic certification, grass‐fed cow milk
SY‐JZ‐LDB	Sanyuan Jizhi	A2β‐casein full‐fat pasteurized (72°C–85°C, 15 s), 7‐day shelf life, 3.5% protein	Bottle	20240603	A2β‐casein‐enriched milk source
JLB‐YXH0.09	Junlebao Yuexianhuo	INF 0.09 s ultra‐instant sterilization (135°C–150°C), 7‐day shelf life, 3.6% protein	Bottle	20240604	Ultra‐high temperature short‐time (UHTST) processing
JLB‐STD	Junlebao	Full‐fat pasteurized (72°C, 15 s), 19‐day shelf life, 3.3% protein	Bottle	20240605	Conventional pasteurization, standard milk source
YL‐STD	Yili	Full‐fat pasteurized (72°C, 15 s), 15‐day shelf life, 3.2% protein	Bottle	20240606	Conventional pasteurization, microfiltration, standard milk source
MN‐ORG	Mengniu	Full‐fat pasteurized (72°C–85°C, 15 s), 15‐day shelf life, 3.6% protein	Bottle	20240607	Organic certification, grass‐fed cow milk
GM‐STD	Guangming Youbei	Full‐fat pasteurized (75°C, 15 s), 15‐day shelf life, 3.6% protein	Bottle	20240608	Conventional pasteurization, local milk source
Product: Plain yoghurt					
SY‐B	Sanyuan	No added sucrose (sweetened with stevia)	Pouch	20240609	Sugar‐free, protein content (2.7 ± 0.1 g/100 g), low calorie (35 kcal/100 g)
SY‐Y	Sanyuan	Original (sucrose: (6.0–8.5) ± 0.2 g/100 g)	Pouch	20240610	Full‐sugar, standard formulation, protein content (2.7 ± 0.1 g/100 g)
JLB‐Q	Junlebao	Reduced sucrose (25% less: 5.2 ± 0.2 g/100 g)	Bottle	20240611	Probiotic‐enriched, low‐sugar, protein content (2.8 ± 0.1 g/100 g)
GM‐Y	Guangming Gulong	Full‐sugar (sucrose: (6.0–8.5) ± 0.2 g/100 g)	Pouch	20240612	Probiotic‐enriched, full‐sugar, high protein (3.3 ± 0.1 g/100 g)
YL‐Y	Yili	Original (sucrose: (6.0–8.5) ± 0.2 g/100 g)	Carton	20240613	Added stabilizers (pectin: 0.15 g/100 g), low‐fat (1.4 ± 0.1 g/100 g), protein content (2.7 ± 0.1 g/100 g)
MN‐Y	Mengniu	Full‐sugar (sucrose: (6.0–8.5) ± 0.2 g/100 g)	Bottle	20240614	Protein content (2.7 ± 0.1 g/100 g), creamy texture

Samples were selected based on a preliminary market survey of top‐selling products in Beijing supermarkets, representing the most common processing methods and formulation types in the Chinese market. For milk, this included different sterilization techniques (pasteurization vs. UHTST) and milk sources (organic, A2β‐casein, conventional). For yoghurt, samples were chosen to cover a range of sugar contents (full‐sugar, reduced, sugar‐free) and brand‐specific formulations. While not exhaustive, this set was designed to capture the key variables of interest for this pilot study.

#### 2.1.2 Sample Pretreatment

Milk samples were equilibrated to 25°C and analyzed directly. Yoghurt samples were diluted 1:1 (w/w) with ultrapure water and homogenized using a high‐speed homogenizer at 4000 rpm for 10 min. The mixture was then centrifuged at 8000×g for 15 min at 4°C. The supernatant was carefully collected and filtered through a 0.45 μm cellulose acetate membrane filter to remove particulate matter and ensure matrix uniformity. The filtrate was subsequently used for electronic tongue analysis (Pan et al. 2019).

### 2.2 Electronic Tongue Analysis

Taste profiles were obtained using the INSENT TS‐5000Z E‐tongue system (INSENT Co. Ltd., Japan) with sensors for sourness (CA0), bitterness (C00), astringency (AE1), umami (AAE), and saltiness (CT0), measuring both initial taste and aftertaste (CPA values). In addition to the basic tastes, the system also provides indices for “Richness” (derived from the umami sensor's aftertaste measurement, reflecting mouthfulness) and “Sweetness” (calculated from sensor responses to sugars, though sweetness is not a direct sensor output in this system and is estimated based on known interaction patterns). A 30 mM KCl + 0.3 mM tartaric acid solution served as the reference (Maniruzzaman and Douroumis 2015; Zhao et al. 2024). Each sample was analyzed in quadruplicate, and taste units (TU) were calculated based on the Weber‐Fechner law—which describes the logarithmic relationship between stimulus intensity and perceived sensation—to transform sensor outputs into values that correlate with human gustatory perception (Zhang et al. 2023). Aftertaste values (Aftertaste‐A for astringency persistence and Aftertaste‐B for bitterness persistence) are calculated from the change in sensor output before and after rinsing with reference solution, as per the instrument's standard protocol (Kobayashi et al. 2010).

### 2.3 Sensory Evaluation

A trained panel (*N* = 12) evaluated the samples in standardized sensory booths using Quantitative Descriptive Analysis (QDA) (Dias et al. 2020; Zhi et al. 2016). Attributes for milk included milk flavor intensity, creaminess, sweetness, thickness, and aftertaste persistence. Yoghurt attributes were sourness, creaminess, thickness, astringency, bitterness, and aftertaste persistence, all rated on a 0–15 unstructured line scale. The 12‐member panel underwent 20 h of training over 4 weeks, using reference standards for each attribute (e.g., skim milk for “milk flavor intensity,” sucrose solutions for “sweetness”). Inter‐panelist consistency, assessed by the Intraclass Correlation Coefficient (ICC, two‐way mixed‐effects model, absolute agreement, average measures), was > 0.85 for all attributes, indicating good agreement.

### 2.4 Chemical Analysis

To explain E‐tongue responses, key chemical components were analyzed for representative samples: Fatty acid composition: Determined via GC–MS (Agilent 7890A‐5975C) after methyl esterification (AOAC method 996.06). Column: DB‐23 (60 m × 0.25 mm × 0.25 μm); carrier gas: He (1 mL/min); temperature program: 100°C (2 min) → 240°C (10 min) at 4°C/min (Chi et al. 2023; Kalit et al. 2014). Hydrophobic peptide content: Determined via reversed‐phase high‐performance liquid chromatography (RP‐HPLC, Agilent 1260) with a C18 column (250 mm × 4.6 mm × 5 μm). Mobile phase: A (0.1% TFA in water), B (0.1% TFA in acetonitrile); gradient: 5% B → 60% B (30 min); detection wavelength: 220 nm (Murray et al. 2017; Newman et al. 2015).

For GC–MS analysis of fatty acids, methyl nonadecanoate (C19:0) was used as an internal standard. A seven‐point calibration curve (*R* ^2^ > 0.99) was constructed for each fatty acid methyl ester. The limit of detection (LOD) and limit of quantification (LOQ) were 0.05 and 0.15 μg/mL, respectively. For RP‐HPLC analysis of hydrophobic peptides, a tryptic digest of β‐casein was used as a reference standard. Recovery rates, determined by spiking samples with known concentrations of a standard peptide, ranged from 92% to 105%.

δ‐Decalactone was quantified simultaneously during the same GC–MS run using selective ion monitoring (SIM). The following ions were monitored: m/z 99 (quantification ion) and m/z 85, 142 (confirmation ions). Methyl nonadecanoate (C19:0) served as the internal standard. A seven‐point calibration curve (*R* ^2^ > 0.99) was constructed using δ‐decalactone standard (purity ≥ 98%, Sigma‐Aldrich, USA) over a concentration range of 0.5–5.0 μg/kg. The limit of detection (LOD) and limit of quantification (LOQ) were 0.05 and 0.15 μg/kg, respectively. Recovery rates, determined by spiking samples with 1.0 μg/kg δ‐decalactone, ranged from 88% to 96%.

### 2.5 Data Analysis

Data are presented as mean ± standard deviation. One‐way ANOVA with Tukey's HSD test (*p* < 0.05) identified significant differences. PCA was performed to visualize sample discrimination. PLSR with sevenfold cross‐validation (SIMCA 14.1 default autofit function) modeled the relationship between E‐tongue data (X‐matrix) and sensory attributes (Y‐matrix). All analyses were conducted using SIMCA‐P+ (v14.1), XLSTAT (v2022.1), and OriginPro 2023. Prior to parametric testing, the Shapiro–Wilk test was used to assess the normality of data distribution, and Levene's test was used to verify the homogeneity of variances. All data met the assumptions for parametric analysis (*p* > 0.05).

## 3 Results

### 3.1 Electronic Tongue Discrimination of Dairy Products

#### 3.1.1 Fresh Milk: Key Taste Variations and Chemical Correlates

The E‐tongue analysis revealed distinct taste profiles across all fresh milk samples. A notable finding was that the recorded sourness values, ranging from −25.95 to −25.59 TU, fell below the reference solution's tasteless threshold (−13 TU), indicating that the sour taste intensity was below the detectable threshold of the instrument under these conditions. In contrast, all other taste indices provided quantifiable responses above their detection limits, with significant inter‐sample variations observed particularly in bitterness, astringency, and saltiness (*p* < 0.05; Table 2).

**TABLE 2:** E‐tongue taste indices of fresh milk samples (mean ± SD, *N =* 4, TU; CV < 5%).

Sample code	Sourness	Bitterness	Astringency	Aftertaste‐B	Aftertaste‐A	Umami	Richness	Saltiness	Sweetness
SY‐milk	−25.72 ± 0.08	9.84 ± 0.02^b^	2.68 ± 0.02^b^	2.72 ± 0.02^ab^	0.13 ± 0.01^c^	9.54 ± 0.02^ab^	11.98 ± 0.04^a^	0.44 ± 0.01^d^	3.19 ± 0.01^a^
SY‐JZ‐ORG	−25.59 ± 0.07	10.77 ± 0.04^a^	4.60 ± 0.02^a^	2.90 ± 0.02^a^	0.73 ± 0.02^a^	9.54 ± 0.02^ab^	11.82 ± 0.03^ab^	1.13 ± 0.02^a^	3.16 ± 0.01^a^
SY‐JZ‐LDB	−25.92 ± 0.09	9.65 ± 0.03^bc^	2.75 ± 0.02^b^	2.99 ± 0.03^a^	0.10 ± 0.01^c^	9.68 ± 0.02^a^	11.78 ± 0.04^ab^	0.88 ± 0.01^b^	3.17 ± 0.01^a^
JLB‐YXH0.09	−25.90 ± 0.08	9.50 ± 0.02^c^	2.67 ± 0.02^b^	2.60 ± 0.02^b^	0.08 ± 0.01^c^	9.75 ± 0.04^a^	11.83 ± 0.02^ab^	0.53 ± 0.02^c^	3.17 ± 0.01^a^
JLB‐STD	−25.85 ± 0.07	9.58 ± 0.02^bc^	2.70 ± 0.01^b^	2.66 ± 0.02^ab^	0.12 ± 0.01^c^	9.71 ± 0.02^a^	11.88 ± 0.02^ab^	0.49 ± 0.01^cd^	3.19 ± 0.01^a^
YL‐STD	−25.95 ± 0.10	9.54 ± 0.03^c^	2.72 ± 0.03^b^	2.64 ± 0.03^ab^	0.13 ± 0.02^c^	9.65 ± 0.03^ab^	11.85 ± 0.03^ab^	0.51 ± 0.02^c^	3.16 ± 0.01^a^
MN‐ORG	−25.68 ± 0.09	10.26 ± 0.03^a^	4.18 ± 0.02^a^	2.84 ± 0.02^a^	0.65 ± 0.02^a^	9.62 ± 0.03^ab^	11.91 ± 0.03^ab^	1.02 ± 0.02^a^	3.14 ± 0.02^a^
GM‐STD	−25.82 ± 0.08	9.76 ± 0.02^b^	2.78 ± 0.02^b^	2.71 ± 0.01^ab^	0.16 ± 0.01^b^	9.63 ± 0.03^ab^	11.95 ± 0.03^a^	0.46 ± 0.01^cd^	3.19 ± 0.02^a^

*Note:* In the same column, different lowercase letters indicate significant differences (*p* < 0.05, Tukey's HSD test).

The organic milk sample (SY‐JZ‐ORG) emerged with the most pronounced taste characteristics, demonstrating significantly elevated bitterness (10.77 ± 0.04 TU) and astringency (4.60 ± 0.02 TU) compared to the ultra‐instant sterilized milk (JLB‐YXH0.09; bitterness = 9.50 ± 0.02 TU, astringency = 2.67 ± 0.02 TU; *p* < 0.05). Chemometric analysis established that this intensified taste profile correlated strongly with SY‐JZ‐ORG's substantially higher content of specific chemical constituents compared to conventional milk: α‐linolenic acid (1.25 ± 0.08 g/100 g fat; see Table S1) and hydrophobic peptides (2.15 ± 0.12 mg/mL; see Table S2), both significantly exceeding levels found in conventional milk (*p* < 0.05). These compounds enhance the response of the E‐tongue's bitterness (C00) and astringency (AE1) sensors through hydrophobic interactions and subsequent membrane potential modulation (Jiang et al. 2018; Wu et al. 2020; Yuan et al. 2023).

Further investigation into SY‐JZ‐ORG's sensory characteristics revealed a 23.7% higher concentration of δ‐decalactone (1.72 versus 1.39 μg/kg in conventional SY‐milk; see Table S1), a key lactone contributing to creamy aroma. While primarily influencing orthonasal perception, these volatile compounds also modulate oral somatosensation, potentially reinforcing “richness” perception and indirectly amplifying the bitterness and astringency signals captured by the E‐tongue (Mu et al. 2021; Stamatopoulos et al. 2014; Kobayashi et al. 2010; Nath et al. 2022). This interpretation aligns with SY‐JZ‐ORG's superior sensory panel scores for “milk flavor intensity” (11.5 ± 1.4) and “thickness” (10.8 ± 1.6) on a 0–15 scale (*p* < 0.05; Table 4).

Aftertaste analysis revealed distinctive temporal patterns across samples. Aftertaste‐A (astringency persistence) exhibited the most considerable variation (0.08–0.70 TU), with SY‐JZ‐ORG showing the most prolonged intensity (0.70 ± 0.08 TU)—8.8‐fold higher than JLB‐YXH0.09 (0.08 ± 0.01 TU, *p* < 0.05). This extended astringency persistence is mechanistically consistent with the slow oral clearance of α‐linolenic acid and hydrophobic peptides, which would prolong interaction with the AE1 sensor (Kobayashi et al. 2010; Gibbins and Carpenter 2013; Zhao et al. 2020). Conversely, Aftertaste‐B (bitterness persistence) demonstrated relative stability across samples (2.57–2.96 TU), suggesting this parameter is less susceptible to variations induced by processing conditions or milk source differences.

#### 3.1.2 Plain Yoghurt: Key Taste Variations and Formulation Effects

**FIGURE 1:**
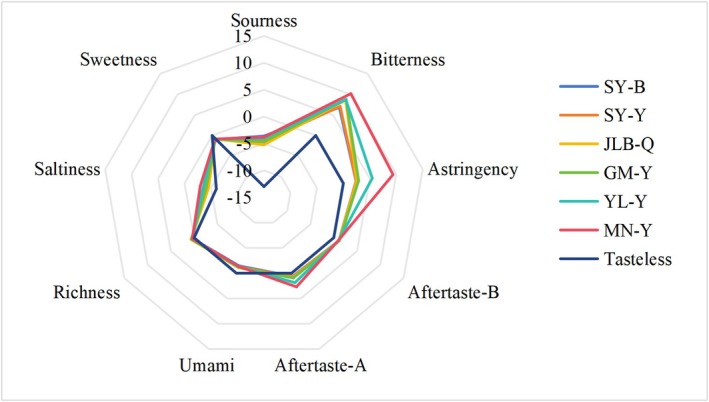
Radar chart of yoghurt based on reference solution (showing taste index differences).

Compared with fresh milk samples, yoghurt exhibited significantly larger inter‐sample variations in taste attributes (*p* < 0.01), with the most pronounced differences observed in astringency and bitterness (Figure 1, Table 3). Notably, Mengniu full‐sugar yoghurt (MN‐Y) had the highest bitterness (10.16 ± 0.16 TU, CV = 1.6%) and astringency (9.38 ± 0.20 TU, CV = 2.1%), which were significantly higher than those of Sanyuan sugar‐free yoghurt (SY‐B: bitterness 6.84 ± 0.10 TU, astringency 2.40 ± 0.08 TU, *p* < 0.01). This phenomenon was attributed to MN‐Y's formulation characteristics: during fermentation, its protein served as a substrate for microbial protease‐mediated hydrolysis, generating bitter‐tasting peptides, while sucrose promoted protein aggregation and interactions with salivary proteins—processes well‐documented to enhance astringency perception (Nath et al. 2022; Lin et al. 2016; Obreque‐Slier et al. 2010).

**TABLE 3:** E‐tongue taste indices of plain yoghurt samples (mean ± SD, *N =* 4, TU; CV < 5%).

Sample code	Sourness	Bitterness	Astringency	Aftertaste‐B	Aftertaste‐A	Umami	Richness	Saltiness	Sweetness
SY‐B	−3.62 ± 0.15^a^	6.84 ± 0.10^d^	2.40 ± 0.08^d^	1.12 ± 0.05^a^	0.63 ± 0.06^c^	−1.44 ± 0.05^a^	0.34 ± 0.04^c^	−3.66 ± 0.12^b^	−0.96 ± 0.05^a^
SY‐Y	−4.54 ± 0.16^b^	7.07 ± 0.11^cd^	2.43 ± 0.07^d^	1.12 ± 0.05^a^	0.68 ± 0.05^c^	−1.29 ± 0.06^ab^	0.46 ± 0.04^bc^	−3.89 ± 0.13^bc^	−0.86 ± 0.04^a^
JLB‐Q	−5.21 ± 0.18^c^	8.64 ± 0.13^b^	2.55 ± 0.09^d^	1.14 ± 0.06^a^	0.88 ± 0.07^bc^	−1.10 ± 0.07^b^	0.65 ± 0.05^a^	−4.54 ± 0.14^d^	−0.81 ± 0.03^a^
GM‐Y	−4.68 ± 0.17^b^	8.78 ± 0.12^b^	2.89 ± 0.09^c^	1.16 ± 0.07^a^	0.95 ± 0.08^b^	−1.18 ± 0.06^ab^	0.58 ± 0.05^ab^	−4.12 ± 0.11^cd^	−0.83 ± 0.04^a^
YL‐Y	−4.04 ± 0.15^b^	8.70 ± 0.12^b^	5.47 ± 0.15^b^	1.02 ± 0.04^a^	1.87 ± 0.12^b^	−1.27 ± 0.08^ab^	0.39 ± 0.04^bc^	−3.54 ± 0.10^b^	−0.89 ± 0.05^a^
MN‐Y	−3.81 ± 0.13^ab^	10.16 ± 0.16^a^	9.38 ± 0.20^a^	1.00 ± 0.05^a^	2.70 ± 0.15^a^	−1.32 ± 0.07^ab^	0.40 ± 0.05^bc^	−2.96 ± 0.09^a^	−0.84 ± 0.03^a^

*Note:* In the same column, different lowercase letters indicate significant differences (*p* < 0.05, Tukey's HSD test).

For sourness—a hallmark sensory attribute of fermented dairy products—the values ranged from −5.21 ± 0.18 TU (JLB‐Q, reduced‐sugar yoghurt) to −3.62 ± 0.15 TU (SY‐B, sugar‐free yoghurt). The lower sourness of JLB‐Q was likely associated with its reduced sucrose content: limited fermentable carbohydrates slowed the metabolic activity of lactic acid bacteria, thereby decreasing lactic acid production (Aktar 2022; Suharman et al. 2021). In terms of saltiness, MN‐Y again showed the highest value (−2.96 ± 0.09 TU, CV = 3.0%), whereas JLB‐Q had the lowest (−4.54 ± 0.14 TU, CV = 3.1%). Consistent with the fresh milk results, yoghurt saltiness exhibited a strong positive correlation with sensory thickness (*r =* 0.83, *p* < 0.01), which further confirmed the role of the E‐tongue's CT0 sensor in indirectly assessing mouthfeel‐related attributes.

In contrast, umami and richness showed minimal variability across all yoghurt samples: umami values ranged from −1.44 to −1.10 TU (CV = 2.8%–4.5%), while richness values varied between 0.34 and 0.65 TU (CV = 5.9%–14.7%). This low variability suggested that the fermentation process, as well as differences in sugar content (full‐sugar, reduced‐sugar, sugar‐free), had no significant impact on the content of umami‐active compounds (e.g., glutamic acid) or the factors contributing to richness perception (e.g., short‐chain fatty acids).

### 3.2 PCA for Sample Discrimination

#### 3.2.1 Fresh Milk: Separation by Milk Source and Sterilization

**FIGURE 2:**
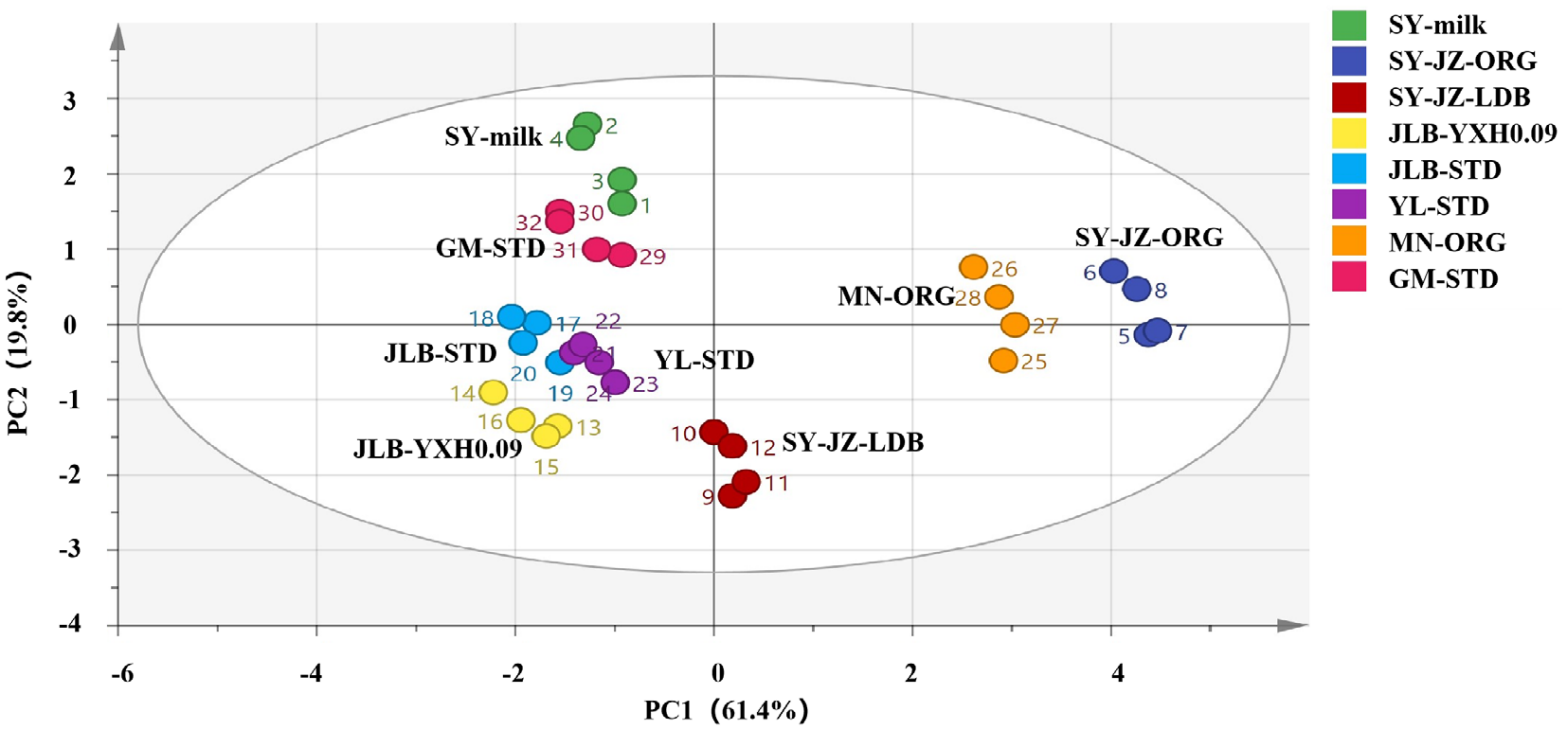
PCA biplot of fresh milk samples based on E‐tongue indices. Symbols represent samples (*N =* 4, error bars represent standard deviation (SD)); 95% confidence ellipses are shown. PC1 = 61.4%, PC2 = 19.8%; cumulative variance = 81.2%.

PCA of fresh milk samples showed distinct clustering based on multifactors (Figure 2). The first two principal components (PC1 and PC2) explained 81.2% of cumulative variance (PC1 = 61.4%, PC2 = 19.8%). Astringency (loading: 0.812) and bitterness (loading: 0.481) were the primary drivers of PC1, separating organic milk samples (SY‐JZ‐ORG, MN‐ORG) from conventional milk on the positive PC1 axis. Aftertaste‐B (loading: 0.623) and saltiness (loading: 0.672) drove PC2, distinguishing ultra‐instant sterilized milk (JLB‐YXH0.09) and A2β‐casein milk (SY‐JZ‐LDB).

To statistically validate these observed groupings, PERMANOVA was performed on the Euclidean distance matrix derived from E‐tongue taste indices. The analysis confirmed significant differences between organic and conventional milk samples (*p* = 0.012) and between pasteurized and UHTST milk samples (*p* = 0.024). For yoghurt, a significant difference was also detected between full‐sugar and sugar‐free samples (*p* = 0.008). These results provide statistical support for the clustering patterns observed in the PCA biplots.

#### 3.2.2 Yoghurt: Separation by Sugar Content and Brand

**FIGURE 3:**
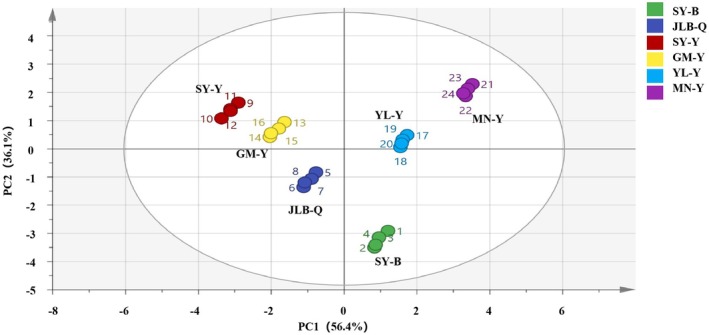
PCA biplot of plain yoghurt samples based on E‐tongue indices. Symbols represent samples (*N =* 4, error bars represent standard deviation (SD)); 95% confidence ellipses are shown. PC1 = 56.4%, PC2 = 36.1%; cumulative variance = 92.5%.

PCA of yoghurt samples achieved strong discrimination, with PC1 and PC2 explaining 92.5% of cumulative variance (PC1: 56.4%, PC2: 36.1%; Figure 3). Astringency (loading: 0.898) was the dominant contributor to PC1, separating full‐sugar yoghurts (high astringency) from low‐sugar/sugar‐free ones. Bitterness (loading: 0.742) and sourness (loading: −0.525) drove PC2, distinguishing samples by sugar content and brand‐specific formulations.

### 3.3 Correlation Among E‐Tongue, Sensory, and Chemical Data

#### 3.3.1 | Fresh Milk: Strong Tripartite Correlations

Sensory scores confirmed E‐tongue findings, with SY‐JZ‐ORG showing the highest “milk flavor intensity” (11.5 ± 1.4) and “thickness” (10.8 ± 1.6) (*p* < 0.05; Table 4). Pearson correlation coefficients were calculated using the mean values of E‐tongue indices and sensory scores for each sample to assess the strength of linear relationships between instrumental and sensory data. Strong positive correlations were observed:

**TABLE 4:** Sensory scores of fresh milk samples (mean ± SD, *N =* 12, 0–15 scale).

Sample code	Milk flavor intensity	Creaminess	Sweetness	Thickness	Aftertaste persistence
SY‐milk	8.2 ± 1.1^c^	7.8 ± 1.3^bc^	6.9 ± 1.0^a^	7.5 ± 1.2^c^	6.3 ± 1.1^c^
SY‐JZ‐ORG	11.5 ± 1.4^a^	10.2 ± 1.5^a^	7.1 ± 1.2^a^	10.8 ± 1.6^a^	9.6 ± 1.4^a^
SY‐JZ‐LDB	8.8 ± 1.2^bc^	8.5 ± 1.4^b^	7.0 ± 1.1^a^	8.1 ± 1.3^bc^	7.2 ± 1.2^bc^
JLB‐YXH0.09	7.1 ± 1.0^d^	6.9 ± 1.1^c^	7.3 ± 1.1^a^	6.7 ± 1.0^d^	5.8 ± 0.9^d^
JLB‐STD	7.8 ± 1.1^cd^	7.2 ± 1.2^bc^	7.2 ± 1.0^a^	7.0 ± 1.1^cd^	6.1 ± 1.0^cd^
YL‐STD	7.5 ± 1.0^d^	7.0 ± 1.1^c^	7.1 ± 1.2^a^	6.8 ± 1.0^d^	5.9 ± 1.0^d^
MN‐ORG	10.8 ± 1.3^a^	9.8 ± 1.4^a^	7.0 ± 1.1^a^	10.2 ± 1.5^a^	9.1 ± 1.3^a^
GM‐STD	8.5 ± 1.2^bc^	7.6 ± 1.2^bc^	7.2 ± 1.0^a^	7.3 ± 1.1^c^	6.5 ± 1.1^c^

*Note:* In the same column, different lowercase letters indicate significant differences (*p* < 0.05, Tukey's HSD test).

E‐tongue bitterness versus “milk flavor intensity”: *r =* 0.958 (*p* < 0.01) and versus hydrophobic peptide content: *r =* 0.959 (*p* < 0.01); E‐tongue astringency vs. “thickness”: *r =* 0.965 (*p* < 0.01) and versus α‐linolenic acid content: *r =* 0.964 (*p* < 0.01); E‐tongue Aftertaste‐A versus “aftertaste persistence”: *r =* 0.955 (*p* < 0.01).

#### 3.3.2 Yoghurt: E‐Tongue Predicts Sensory Mouthfeel

MN‐Y had the highest “astringency” (11.2 ± 1.7, 0–15 scale) and “thickness” (12.5 ± 1.8, 0–15 scale) (*p* < 0.01; Table 5), consistent with its extreme E‐tongue astringency (9.38 TU). Key correlations included:

**TABLE 5:** Sensory scores of plain yoghurt samples (mean ± SD, *N =* 12, 0–15 scale).

Sample code	Sourness	Creaminess	Thickness	Astringency	Bitterness	Aftertaste persistence
SY‐B	11.2 ± 0.5^a^	6.8 ± 1.1^c^	7.1 ± 1.2^d^	5.9 ± 1.0^d^	4.2 ± 0.8^c^	6.5 ± 1.1^d^
SY‐Y	9.8 ± 1.3^b^	7.5 ± 1.2^bc^	7.6 ± 1.1^cd^	6.2 ± 1.0^d^	4.8 ± 0.9^bc^	7.0 ± 1.1^cd^
JLB‐Q	7.5 ± 1.1^d^	8.9 ± 1.4^a^	8.3 ± 1.3^c^	6.8 ± 1.1^cd^	5.8 ± 1.0^b^	7.8 ± 1.2^c^
GM‐Y	8.2 ± 1.2^c^	8.5 ± 1.3^ab^	8.9 ± 1.4^bc^	7.2 ± 1.1^c^	6.1 ± 1.1^b^	8.2 ± 1.3^bc^
YL‐Y	9.2 ± 1.3^bc^	8.6 ± 1.4^ab^	10.2 ± 1.5^b^	9.8 ± 1.6^b^	6.8 ± 1.2^b^	9.1 ± 1.4^b^
MN‐Y	10.5 ± 1.4^a^	9.8 ± 1.5^a^	12.5 ± 1.8^a^	11.2 ± 1.7^a^	8.5 ± 1.4^a^	10.8 ± 1.6^a^

*Note:* In the same column, different lowercase letters indicate significant differences (*p* < 0.05, Tukey's HSD test).

E‐tongue astringency versus “thickness”: *r =* 0.965 (*p* < 0.01) and versus protein content: *r =* −0.70 (*p* > 0.01); E‐tongue sourness versus “sourness”: *r =* 0.910 (*p* < 0.05) and versus acidity: *r =* 0.961 (*p* < 0.01); E‐tongue Aftertaste‐A versus “aftertaste persistence”: *r =* 0.967 (*p* < 0.01). The overall negative correlation between protein content and astringency across all yoghurt samples (*r* = −0.70) was unexpected and may be influenced by the specific formulation of certain samples (e.g., the presence of stabilizers like pectin in YL‐Y, which can modulate astringency perception). Notably, the sample with the highest astringency (MN‐Y, 9.38 TU) did not have the highest protein content (2.7/100 g), indicating that factors beyond total protein—such as the degree of proteolysis and the presence of sucrose—play a dominant role in driving astringency. Further investigation with a larger sample set is needed to clarify these relationships.

### 3.4 PLSR Models: Predictive Power for Sensory Attributes

Both the fresh milk and yoghurt PLSR models were constructed using SIMCA 14.1 with the default autofit function, which applies sevenfold cross‐validation. For the yoghurt dataset (*N =* 6), this is mathematically equivalent to leave‐one‐out cross‐validation (LOOCV)—the most appropriate validation method when sample size is extremely limited, as it maximizes the use of training data. Variable Importance in Projection (VIP) scores were used to identify the most influential E‐tongue indices in the PLSR models.

Fresh milk model: Explained 97.1% of sensory variance (R^2^Y = 0.971) and had 85.6% predictive power (Q^2^ = 0.856). Bitterness (VIP = 1.13), astringency (VIP = 1.13), and aftertaste‐A (VIP = 1.12) were the top predictors (Tables 6 and 7).

**TABLE 6:** Performance of PLSR models for E‐tongue‐sensory correlation.

Model	Predictors (X)	Responses (Y)	R^2^X	R^2^Y	Q^2^	Top 3 predictors
Fresh milk	8 E‐tongue indices	5 sensory attributes	0.999	0.971	0.856	Bitterness (1.13), Astringency (1.13), Aftertaste‐A (1.12)
Plain yoghurt	9 E‐tongue indices	6 sensory attributes	0.962	0.972	0.902	Bitterness (1.27), Astringency (1.24), Aftertaste‐A (1.27)

*Note:* Both PLSR models were validated using sevenfold cross‐validation (SIMCA 14.1 default). For the yoghurt model (*N =* 6), this is equivalent to leave‐one‐out cross‐validation (LOOCV).

**TABLE 7:** VIP values of E‐tongue indices in PLSR models.

E‐tongue index	Fresh milk (VIP)	Plain yoghurt (VIP)
Bitterness	1.13	1.27
Astringency	1.13	1.24
Aftertaste‐A	1.12	1.27
Aftertaste‐B	1.04	1.06
Umami	0.95	0.75
Richness	0.57	0.69
Sourness	—	0.70
Saltiness	1.09	0.94
Sweetness	0.85	0.83

Yoghurt model: Explained 97.2% of sensory variance (R^2^Y = 0.972) and had 90.2% predictive power (Q^2^ = 0.902). Astringency (VIP = 1.24), bitterness (VIP = 1.27), and aftertaste‐A (VIP = 1.27) were the top predictors (Tables 6 and 7).

For both models, residual analysis confirmed the absence of outliers (Grubbs test, *p* > 0.05) and normality of distribution (Shapiro–Wilk test, *p* > 0.05).

## 4 Discussion

### 4.1 Multi‐Factor Interactions Shape Dairy Flavor: Evidence From E‐Tongue and Chemistry

This study is the first to systematically explore multifactor interactions (processing + milk source + formulation) on dairy flavor using E‐tongue, supported by chemical analysis. For fresh milk: milk source (organic vs. conventional) was the dominant factor driving flavor differences: organic milk (SY‐JZ‐ORG, MN‐ORG) had 4.27%–12.42% higher bitterness and 54.81%–71.64% higher astringency than conventional milk (SY‐milk, JLB‐STD), attributed to higher α‐linolenic acid (unsaturated fatty acids enhance hydrophobic interactions with C00 sensors) and hydrophobic peptides (bitter compounds derived from casein hydrolysis) (Molkentin and Giesemann 2007; Schwendel, Morel, et al. 2015; Schwendel, Wester, et al. 2015; Galgano et al. 2016).

Sterilization technique interacted with milk source: ultra‐instant sterilization (JLB‐YXH0.09) reduced bitterness by 0.84% vs. pasteurization (JLB‐STD), likely due to minimal protein denaturation (reducing peptide release) and suppressed Maillard reaction (Alais et al. 1978; Clare et al. 2005).

For yoghurt: sugar content interacted with brand: full‐sugar yoghurts (MN‐Y, GM‐Y) had 20.40%–290.80% higher astringency than low‐sugar/sugar‐free counterparts (SY‐B, JLB‐Q), as sucrose promotes protein aggregation (enhancing AE1 sensor response) (Miele et al. 2017; Miyashita and Etoh 2013).

Brand‐specific formulations (e.g., protein content, stabilizers) had a substantial impact. Notably, MN‐Y (full‐sugar, 2.7/100 g protein) showed 290.80% higher astringency than SY‐B (sugar‐free, 2.7/100 g protein), even though both had comparable protein levels. This highlights the dominant role of sugar content in promoting protein aggregation and enhancing astringency, as well as the influence of other formulation factors such as stabilizers. Interestingly, the sample with the highest protein content (GM‐Y, 3.3/100 g) did not exhibit proportionally high astringency (2.89 TU), further supporting that astringency in yoghurt is primarily driven by proteolysis and sugar‐induced protein aggregation rather than total protein content alone.

The higher bitterness and astringency in organic milk (SY‐JZ‐ORG, MN‐ORG) can be mechanistically linked to its distinct chemical composition. Elevated levels of α‐linolenic acid, a polyunsaturated fatty acid, enhance hydrophobic interactions with the E‐tongue's lipid membranes, potentiating the sensor response for bitterness and astringency. Concurrently, higher hydrophobic peptide content, likely resulting from endogenous proteolysis, directly contributes to bitterness. In yoghurt, the pronounced astringency in full‐sugar samples (e.g., MN‐Y) is attributed to the ability of sucrose to promote protein aggregation, which in turn enhances the interaction of proteins with salivary proline‐rich proteins and the AE1 sensor.

While the strong correlations (*r* > 0.95) between α‐linolenic acid, hydrophobic peptides, and the corresponding E‐tongue indices suggest a key role for these compounds in driving bitterness and astringency, we acknowledge that these relationships are correlational, not causal. The observed flavor profiles are likely the result of a complex mixture of multiple compounds. Further studies involving targeted manipulation experiments, such as spiking conventional milk with these compounds, are needed to establish definitive causal links.

### 4.2 E‐Tongue as a Reliable Tool: Advantages Over Traditional Methods

The E‐tongue addressed key limitations of traditional approaches:

*Objectivity:* Unlike sensory evaluation (inter‐panelist CV = 9.5%–14.2%), E‐tongue data showed low variability (CV < 5%), ensuring consistency across batches.

*Mechanistic interpretability:* By correlating E‐tongue sensor responses with specific chemical components (e.g., bitterness vs. hydrophobic peptides, *r =* 0.959), this study provides a more mechanistic interpretation of the instrument's output, moving beyond the purely empirical “black box” approach that has characterized some previous E‐tongue research where flavor differences were identified without explaining their chemical basis (Nath et al. 2022; Chang et al. 2008).

*Predictive power:* PLSR models (R^2^Y = 0.971–0.972, Q^2^ = 0.856–0.902) enabled rapid sensory prediction without trained panels—critical for high‐throughput quality control (e.g., predicting “thickness” from astringency index).

Table 8 compares this study with existing dairy E‐tongue research, highlighting its innovation in multi‐factor exploration and mechanistic validation.

**TABLE 8:** Comparison of this study with existing dairy E‐tongue research.

Study	Dairy type	Variables investigated	E‐tongue system	Chemical validation	Innovation gap addressed
Ding et al. (2024)	UHT milk	Brand (single variable)	INSENT TS‐4000	No	Added sterilization + milk source
Meenakshi et al. (2018)	Probiotic yoghurt	Storage time (single variable)	Alpha M.O.S.	No	Added formulation (sugar) + brand
Saccaro et al. (2009)	Cheese	Ripening time (single variable)	INSENT TS‐5000Z	Yes (fatty acids)	Focused on single product; no multifactor
This study	Fresh milk + yoghurt	Sterilization + milk source + sugar + brand	INSENT TS‐5000Z	Yes (fatty acids, peptides)	Systematic multivariable interaction + tripartite correlation (E‐tongue‐sensory‐chemistry)

### 4.3 Practical Implications for Dairy Industry

The findings provide actionable insights for dairy manufacturers:

*Fresh milk optimization:* For organic milk, reduce astringency by adjusting cow feeding (e.g., mixing grass with hay to lower α‐linolenic acid) or using milder sterilization (pasteurization vs. UHT). The perception of astringency varies by market: while some consumers associate it with creaminess and quality, others may find it undesirable. Thus, adjusting astringency levels allows manufacturers to tailor products to regional preferences.

*Yoghurt development:* For sugar‐free products, maintain flavor consistency by adding 0.1% pectin (a known astringency inhibitor) to compensate for reduced sucrose (Hayashi et al. 2005; Carter et al. 2020).

*Quality control:* Use E‐tongue astringency (VIP = 1.24 in yoghurt and 1.13 in milk, see Table 7) as a rapid indicator of mouthfeel attributes to ensure batch consistency, reducing reliance on sensory panels. The findings offer actionable insights for dairy manufacturers. For fresh milk, astringency in organic products can be modulated by adjusting cattle feed (e.g., balancing grass and hay to influence α‐linolenic acid content) or by selecting milder sterilization methods. For yoghurt, the strong correlation between E‐tongue astringency and sensory thickness suggests that the astringency index can serve as a rapid, nonsubjective quality control tool to ensure batch‐to‐batch consistency in mouthfeel, reducing reliance on time‐consuming and variable sensory panels.

### 4.4 Limitations and Future Directions

This study focused on fresh milk and plain yoghurt; future research should expand to other dairy products (e.g., cheese, ice cream) and explore flavor changes during storage (e.g., 4°C for 14 days). Additionally, combining E‐tongue with untargeted metabolomics (e.g., LC–MS/MS) could identify specific bitter/astringent compounds (e.g., casein‐derived peptides), further enhancing mechanistic understanding. Finally, integrating consumer acceptance data (e.g., preference tests) with E‐tongue profiles would bridge objective measurement and market preference.

While the E‐tongue effectively discriminated samples, its performance can be influenced by the complex matrix of dairy products. High fat and protein content may interact with the lipid membranes, potentially interfering with sensor responses. This underscores the necessity of the sample pretreatment steps detailed in Section 2.1.2 to ensure matrix consistency and data reliability.

We acknowledge the limitation of a modest sample size, which can increase the risk of model overfitting in PLSR. To test the robustness of our findings, we supplemented the analysis with simple linear regression models for key relationships. For instance, the strong correlations between astringency and sensory thickness, as well as between hydrophobic peptide content and bitterness (R^2^ = 0.88, *p* < 0.001; Figure S1), were highly consistent with the PLSR trends, supporting the validity of our main conclusions. Nevertheless, larger‐scale studies are needed to confirm these findings.

We recognize that the sample size (*n* = 8 for milk, *n* = 6 for yoghurt) is modest for multivariable analysis and that factors such as lactation stage, precise feed composition, and fermentation parameters were not controlled. Consequently, our findings on multi‐factor interactions should be considered exploratory. This study was designed as a pilot investigation to demonstrate the potential of the integrated E‐tongue‐sensory‐chemistry approach. Future studies with larger, more controlled sample sets are necessary to validate and refine these initial observations.

## 5 Conclusions

This study demonstrates that the INSENT TS‐5000Z E‐tongue, combined with sensory and chemical analyses, can effectively deconvolute complex flavor profiles of dairy products arising from multi‐factor interactions. Astringency emerged as a key discriminating factor for both milk and yoghurt, strongly correlating with specific chemical constituents (e.g., α‐linolenic acid) and sensory mouthfeel attributes. The robust predictive ability of the PLSR models (Q^2^ > 0.85) further validates its utility as an objective, high‐throughput tool for quality control and data‐driven product development in the dairy industry.

## Author Contributions

**Juan Huang:** conceptualization, methodology, formal analysis, investigation, writing – original draft. **Jing‐wei Zhong:** validation, investigation, data curation. **Li‐guang Jiang:** software, validation, visualization. **Yan‐yan Huang:** resources, writing – review and editing. **Dong‐mei Liu:** supervision, project administration, Funding acquisition, writing – review and editing. All authors have read and approved the final manuscript.

## Funding

This study was supported by the National Natural Science Foundation of China (No. 31771908) and the Guangdong Science and Technology Program Key Project (No. 201903010015). The funders had no role in the study design, data collection and analysis, decision to publish, or preparation of the manuscript.

## Ethics Statement

This study was conducted in accordance with the Declaration of Helsinki. All participants provided informed consent prior to sensory evaluation, and the study protocol was approved by the Ethics Committee of the Faculty of Food Science and Engineering, Foshan University (Approval No. SYXK 2020–0235). Ethical approval was obtained before the commencement of the study.

## Conflicts of Interest

The authors declare no conflicts of interest.

## Supporting information


**Figure S1:** Simple linear regression analysis of hydrophobic peptide content versus electronic tongue bitterness value in fresh milk samples (*R* ^2^ = 0.88, *p* < 0.001).
**Table S1:** Composition of key flavor fatty acids and δ‐decalactone in representative fresh milk samples (g/100 g fat, mean ± SD, *N* = 3).
**Table S2:** Hydrophobic peptide content in fresh milk samples (mg/mL, mean ± SD, *N* = 3).

## Data Availability

Data sharing is not applicable to this article as no datasets were generated or analysed during the current study.
