# Supplementary material for: Comprehensive Flavor Profiling of Dairy Products Using Electronic Tongue: Discrimination Based on Processing Parameters and Formulations
Source: Food Sci Nutr. 2026 May 13;14(5):e71781. doi: 10.1002/fsn3.71781 (PMC13169148; doi:10.1002/fsn3.71781)
Supplement: Supplementary file 1 — Figure S1: Simple linear regression analysis of hydrophobic peptide content versus electronic tongue bitterness value in fresh milk samples (R 2 = 0.88, p < 0.001). Table S1: Composition of key flavor fatty acids and δ‐decalactone in representative fresh milk samples (g/100 g fat, mean ± SD, N = 3). Table S2: Hydrophobic peptide content in fresh milk samples (mg/mL, mean ± SD, N = 3). [file FSN3-14-e71781-s001.zip › fsn371781-sup-0002-Tables.docx]

**Supplementary TABLE S1 |** Composition of key flavor fatty acids in representative fresh milk samples (g/100 g fat, mean ± SD, *N*=3).

| **Compound (unit)** | **SY-milk** | **SY-JZ-ORG** | **JLB-YXH0.09** |
| --- | --- | --- | --- |
| **Fatty acid (g/100 g fat)** |  |  |  |
| Butyric acid (C4:0) | 1.52 ± 0.05 ^c^ | 2.01 ± 0.06 ^a^ | 1.68 ± 0.05 ^b^ |
| Hexanoic acid (C6:0) | 1.85 ± 0.06 ^b^ | 2.24 ± 0.07 ^a^ | 1.90 ± 0.06 ^b^ |
| Octanoic acid (C8:0) | 1.08 ± 0.04 ^b^ | 1.31 ± 0.05 ^a^ | 1.10 ± 0.04 ^b^ |
| Decanoic acid (C10:0) | 2.41 ± 0.08 ^b^ | 2.89 ± 0.09 ^a^ | 2.45 ± 0.08 ^b^ |
| Lauric acid (C12:0) | 2.98 ± 0.10 ^ab^ | 3.15 ± 0.10 ^a^ | 2.85 ± 0.09 ^b^ |
| **Total Short-Chain Fatty Acids (C4-C10)** | 8.12 ± 0.25 ^b^ | 10.25 ± 0.31 ^a^ | 8.35 ± 0.26 ^b^ |
| Oleic acid (C18:1) | 22.15 ± 0.65 ^b^ | 25.83 ± 0.78 ^a^ | 21.90 ± 0.66 ^b^ |
| Linoleic acid(C18:2) | 2.85 ± 0.09 ^b^ | 3.22 ± 0.10 ^a^ | 2.81 ± 0.09 ^b^ |
| α-Linolenic acid(C18:3) | 0.68 ± 0.05 ^b^ | 1.25 ± 0.08 ^a^ | 0.71 ± 0.05 ^b^ |
| **Total Key Unsaturated Fatty Acids** | 25.68 ± 0.79 ^b^ | 30.30 ± 0.96 ^a^ | 25.42 ± 0.80 ^b^ |
| **Total Unsaturated Fatty Acids** | 32.45 ± 0.98 ^b^ | 37.56 ± 1.13 ^a^ | 32.10 ± 0.97 ^b^ |
| **Lactone** |  |  |  |
| **δ‑Decalactone (μg/kg)** | 1.39 ± 0.11 ^b^ | 1.72 ± 0.09 ^a^ | 1.41 ± 0.10 ^b^ |

Note: δ‑Decalactone is a lactone (fatty ester) quantified simultaneously during the same GC‑MS run using selective ion monitoring (SIM). Different lowercase letters in the same row indicate significant differences (*p* < 0.05, Tukey’s HSD test).

**Supplementary TABLE S2 |** Hydrophobic peptide content in fresh milk samples (mg/mL, mean ± SD, *N*=3).

| Sample Code | SY-milk | SY-JZ-ORG | SY-JZ-LDB | JLB-YXH0.09 | JLB-STD | YL-STD | MN-ORG | GM-STD |
| --- | --- | --- | --- | --- | --- | --- | --- | --- |
| Hydrophobic peptide content | 1.19 ± 0.08 ^c^ | 2.15 ± 0.12 ^a^ | 1.42 ± 0.09 ^b^ | 1.05 ± 0.07 ^c^ | 1.22 ± 0.08 ^c^ | 1.18 ± 0.07 ^c^ | 1.98 ± 0.11 ^a^ | 1.25 ± 0.08 ^c^ |

Note: In the same line, different lowercase letters indicate significant differences (*p* < 0.05, Tukey’s HSD test).
