# Supplementary figures and images for: Comprehensive Flavor Profiling of Dairy Products Using Electronic Tongue: Discrimination Based on Processing Parameters and Formulations
Source: Food Sci Nutr. 2026 May 13;14(5):e71781. doi: 10.1002/fsn3.71781 (PMC13169148; doi:10.1002/fsn3.71781)

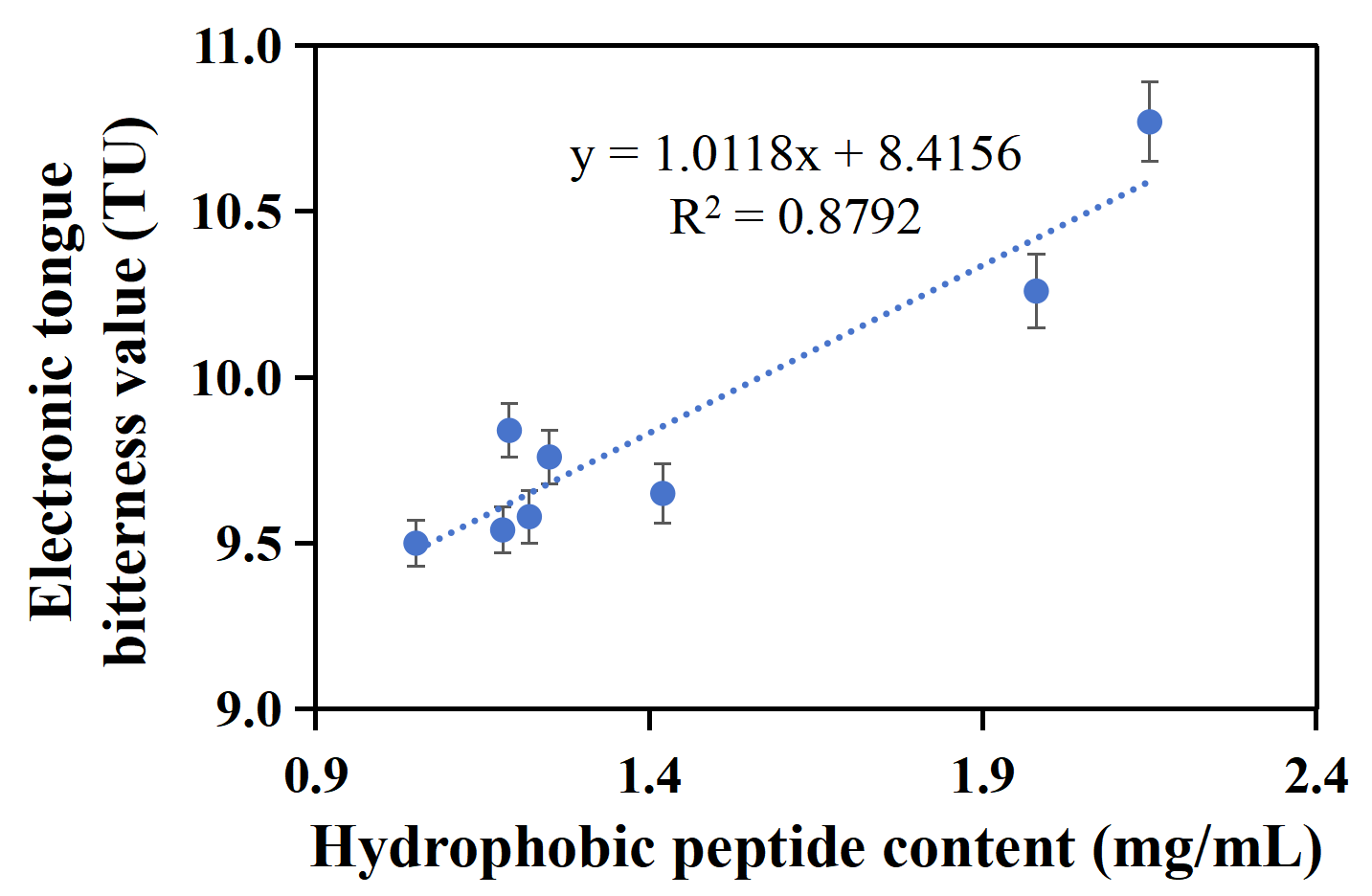

Supplement: Supplementary file 1 — Figure S1: Simple linear regression analysis of hydrophobic peptide content versus electronic tongue bitterness value in fresh milk samples (R 2 = 0.88, p < 0.001). Table S1: Composition of key flavor fatty acids and δ‐decalactone in representative fresh milk samples (g/100 g fat, mean ± SD, N = 3). Table S2: Hydrophobic peptide content in fresh milk samples (mg/mL, mean ± SD, N = 3). [file FSN3-14-e71781-s001.zip › fsn371781-sup-0001-FigureS1.tif]
